# Higher Gravidity and Parity Are Associated with Increased Prevalence of Metabolic Syndrome among Rural Bangladeshi Women

**DOI:** 10.1371/journal.pone.0068319

**Published:** 2013-08-02

**Authors:** Shamima Akter, Subrina Jesmin, Md. Mizanur Rahman, Md. Majedul Islam, Most. Tanzila Khatun, Naoto Yamaguchi, Hidechika Akashi, Taro Mizutani

**Affiliations:** 1 Health & Disease Research Center for Rural Peoples (HDRCRP), 14/15, Probal Housing Ltd., Mohammadpur, Dhaka, Bangladesh; 2 Graduate School of Medicine, Faculty of Medicine, University of Tsukuba, Tsukuba, Ibaraki, Japan; 3 National Center for Global Health and Medicine (NCGM), Toyama, Shinjuku-ku, Tokyo, Japan; 4 Department of Global Health Policy, University of Tokyo, Tokyo, Japan; Iran University of Medical Sciences, Islamic Republic of Iran

## Abstract

**Background:**

Parity increases the risk for coronary heart disease; however, its association with metabolic syndrome among women in low-income countries is still unknown.

**Objective:**

This study investigates the association between parity or gravidity and metabolic syndrome in rural Bangladeshi women.

**Methods:**

A cross-sectional study was conducted in 1,219 women aged 15–75 years from rural Bangladesh. Metabolic syndrome was defined according to the standard NCEP-ATP III criteria. Logistic regression was used to estimate the association between parity and gravidity and metabolic syndrome, with adjustment of potential confounding variables.

**Results:**

Subjects with the highest gravidity (> = 4) had 1.66 times higher odds of having metabolic syndrome compared to those in the lowest gravidity (0-1) (*P*_trend_ = 0.02). A similar association was found between parity and metabolic syndrome (*P*_trend_ = 0.04), i.e., subjects in the highest parity (> = 4) had 1.65 times higher odds of having metabolic syndrome compared to those in the lowest parity (0-1). This positive association of parity and gravidity with metabolic syndrome was confined to pre-menopausal women (*P*_trend_ <0.01). Among the components of metabolic syndrome only high blood pressure showed positive association with parity and gravidity (*P*_trend_ = 0.01 and <0.001). Neither Parity nor gravidity was appreciably associated with other components of metabolic syndrome.

**Conclusions:**

Multi parity or gravidity may be a risk factor for metabolic syndrome.

## Introduction

The prevalence of non-communicable diseases (NCD) has significantly increased world-wide [1], with a sharp rise in low-income countries, where infectious diseases remain a significant problem [2]. This problem is particularly pronounced in South Asian populations, which have a much higher prevalence of type 2 diabetes and cardiovascular disease that occur at an earlier age and is associated with high premature mortalities [3]. Metabolic syndrome is a combination of intermediate risk factors, including obesity, glucose intolerance, insulin resistance, dyslipidemia and hypertension, which in turn predisposes individuals to risks associated with cardiovascular disease and type 2 diabetes by two- to threefold and three- to five fold, respectively [4–6]. Understanding potential relationships of reproductive conditions and history with metabolic syndrome may help to identify and alleviate high-risk individuals and those with early or established diseases.

Pregnancy and child bearing are timed physiological conditions. However, there is some evidence that these two conditions may have a long-term impact on the health of women. For instance, it is generally assumed that pregnancy associated insulin resistance resolves after parturition, but subtle metabolic changes could persist [7–9]. According to a systematic review of epidemiologic evidence, it has been shown that having a high number of reproductive events increases a woman’s risk of developing cardiovascular disease [10]. Nonetheless, evidence regarding the association between reproductive events and the prevalence of diabetes mellitus is inconsistent. For this reason, it is of interest to investigate the association of parity or gravidity with metabolic syndrome.

To date, a few studies have investigated the association between parity and metabolic syndrome and found a positive association between parity and metabolic syndrome [11–14]. However, these studies are mainly from the West [11,12], Middle East [14], and East Asian populations [13]. Surprisingly, there has been no report on this issue from South Asia, where the population is more prone to metabolic syndrome and its prevalence (metabolic syndrome) is currently growing at an alarming rate in this subcontinent [15]. For instance, in Bangladesh more than one third adult women have metabolic syndrome, thus posing a serious emerging public health problem [16,17]. Given the paucity of evidence in South Asian populations and particularly in a low-income country, such as Bangladesh, here, we aimed to investigate the association of parity or gravidity with metabolic syndrome and its components in Bangladeshi women. We hypothesized that higher parity or gravidity is positively associated with metabolic syndrome after controlling for socio-demographic, lifestyle and reproductive factors.

## Data and Methods

### Study Procedure and Subjects

This cross-sectional study is a community-based study conducted on women from rural Bangladesh in 2009-2010. A total of 1535 women aged 15-70 years were selected using the stratified multistage random sampling. We used the World Health Organization’s (WHO) STEPS approach (modified), which entails a stepwise collection of the risk factor data, based on standardized questionnaires covering the following parameters: demographic characteristics, somatic illnesses, somatic and mental symptoms, medications, life style, and health-related behavior (step 1), basic physical measures (step 2) and basic biochemical investigations, such as blood glucose and cholesterol (step 3). The women were recruited from 4 village communities located in Gabindagonj Upazilla (subdistrict) of Gaibandha district. After the communities were picked (division, district, Upazila and villages), the respondents were then selected randomly. The details of the study procedure and study area have been described elsewhere [16,18]. In brief, data from participants were obtained through interviews and clinical examinations at mobile examination centers, where blood samples were also collected. The study was approved by the Ethical Committee of the Health and Disease Research Center of Rural Peoples (HDRCRP), Dhaka, Bangladesh, and conforms to the principles outlined in the Helsinki Declaration. All subjects gave their written informed consent prior to participation. In case of subjects below 18 years of age, a written informed consent was obtained from guardians on the behalf of the young participants involved in this study and the ethics committees approve this consent procedure. Prior to the survey, our enumerator carefully read the consent form to the subject and then very briefly explained the aims and importance of the study. This consent form contained information on the objectives of the study, risks, benefits and freedom of participation, and confidentiality.

Out of the total 1535 women, we excluded subjects with missing information on triglyceride, high-density lipoprotein (HDL) cholesterol and fasting blood glucose. We further excluded subjects with missing information on parity, gravidity, or missing information for any of the covariates used in the main analysis. After all these exclusions, the final number of women who remained in the study was 1219 subjects.

### Anthropometric and Other Variables

Anthropometric measurements on individuals wearing light clothing and without shoes, were conducted by well-trained examiners, as described here: height was measured to the nearest 0.1 cm using the portable stadiometer; weight was measured in an upright position, to the nearest 0.1 kg, using a calibrated balance beam scale; body mass index (BMI) was calculated as the body weight (kg) divided by the square of the body height (m^2^); and waist circumference measurements were taken at the end of normal expiration, to the nearest 0.1 cm, by measuring from the narrowest point between the lower borders of the rib cage and the iliac crest; blood pressure was measured twice in the right arm in a sitting position using the standard mercury manometer and cuff, to the nearest 2 mmHg, with the initial reading taken at least 5 minutes after the subject was made comfortable, and again after an interval of 15 minutes. The average systolic and diastolic blood pressures were then estimated. Tobacco use, marital status, use of contraceptives, menopausal status, and experience of pregnancy or number of live births were self-reported. Ever tobacco users were defined as one who was current tobacco users (smoked/chewed tobacco) and who had not smoked/chewed tobacco in the past 30 days preceding the survey but had tried in the past. In this study, women were categorized as post-menopausal if their last menses was at least 12 months prior to the study; pre-menopausal if they had an unchanged and regular menstrual pattern during the last five years, without typical climacteric complaints.

### Biochemical Analysis

Blood for biochemical analysis was obtained from the participants after a 10-12 hour overnight fast. The blood samples were collected using the standard blood sample collection procedure. Immediately after collection of blood and labeling the blood vials, the samples were transported to the National Center for Global Health and Medicine (NCGM), Japan, for biochemical assessment. For analysis, the serum was immediately separated from the blood by centrifugation for in order to evaluate plasma concentration of lipids. Triglyceride levels were measured by lipoprotein lipase method (Wako Chemicals, Tokyo, Japan), HDL cholesterol was measured with the Determiner-L kit (Kyowa Co Ltd, Tokyo, Japan). Fasting plasma glucose levels were measured with the Hexokinase G-6-PDH kit (Wako Pure Chemical Industries Ltd, Osaka, Japan).

### Assessment of Gravidity or Parity

Our main parameter is gravidity or parity of women. Gravidity is defined as the number of pregnancies, including lost pregnancies, due to stillbirths, whereas parity refers to the number of biological live births. This information was obtained by personal interviews at the time of the survey. These questions were conducted in an open-ended form, e.g., “how many live births have you had?” or “how many times have you been pregnant, including pregnancies that ended up in miscarriage or still births?” For data analysis of the present study, parity or gravidity was categorized into four groups (0-1, 2, 3 and ≥ 4). In the present study only 3.69% of our subjects were nulliparous women. Therefore, we choose to group 0 and 1 live birth into one category and considered this category as the reference or baseline category.

### Definition of Metabolic Syndrome and Risk Factors

Metabolic syndrome and metabolic risk factors were defined according to the standard criteria of the National Cholesterol Education Program’s Adults Treatment Panel III (NCEP-ATP III) [19]. Three or more of the following components constituted metabolic syndrome: a) abdominal obesity, as measured by a waist circumference of ≥ 88 cm for women; b) high fasting blood glucose (≥110 mg/dL or ≥6.1 mmol/L) or patients diagnosed with diabetes; c) high triglycerides (≥150 mg/dL or ≥1.7 mmol/L); d) low HDL cholesterol (<50 mg/dL or <1.29 mmol/L); e) and high blood pressure (≥130/≥85mmHg). Also, participants who at the time of the study reported to be on anti-hypertensive or anti-diabetic medications (insulin or oral agents) were considered to have high blood pressure or high fasting blood glucose, respectively.

### Statistical Analysis

Characteristics of the study participants were presented based on the order of the parity number. Trend association of demographic and reproductive parameters and cardiovascular risk factor by parity were assessed using linear regression analysis for continuous variables or Mantel-Haenszel chi-square test for categorical variables, with ordinal numbers 1-4 assigned to increasing categories of parity.

To evaluate the magnitude of the association of parity and gravidity with metabolic syndrome and its components (obesity, high triglycerides, low HDL cholesterol, high blood pressure, and high fasting blood glucose), we estimated adjusted odds ratio (OR) and 95% confidence interval with multivariable logistic regression models. The first models were unadjusted. The second models were adjusted for age (year, continuous), BMI (kg/m^2^, continuous), education (illiterate, had formal education), marital status (currently married or others), tobacco users (ever or never), use of contraceptives (ever or never), and age at first pregnancy (year, continuous). Similarly, we examined the association between parity and metabolic syndrome and its components, according to the menopausal status (pre-menopause and post-menopause), since menopause is known as an important risk factor for metabolic syndrome. The *P* for interaction was assessed using likelihood ratio test comparing models with or without interaction terms for the interaction between parity and menopausal status. Trend association was assessed by assigning ordinal numbers 1-4 to increasing numbers of parity or gravidity. Two-sided *P* values <0.05 were regarded as statistically significant. All analyses were performed using statistical software STATA version 12.0 (Lakeway Drive, College Station, Texas, USA).

## Results

Table 1, which shows the characteristics of the study subjects based on parity, reveals that subjects with higher parity were more likely to have no formal education and were tobacco users, but less likely to be currently married. Parity was positively associated with current age, fasting blood glucose, triglyceride, HDL cholesterol, systolic blood pressure and diastolic blood pressure.

**Table 1:** Baseline characteristics and cardio-metabolic risk factors by parity.

	Number of live births				
	(0-1)	2	3	≥4	Trend *P* ^b^
N	285	389	281	264	
Age (years)	29.79 ± 11.76^a^	34.27 ± 10.36	41.02 ± 10.3	45.85 ± 11.01	<0.001
Education (illiterate, %)	35.79	43.44	56.94	60.61	<0.001
Ever use of contraceptives (%)	16.84	21.85	19.22	11.74	0.07
Currently married (%)	92.28	92.8	89.32	86.74	0.041
Use of tobacco products (ever, %)	9.12	17.48	19.93	22.73	<0.001
BMI (kg/m^2^)	21.94 ± 3.95	22.39 ± 3.86	22.29 ± 3.91	21.62 ± 4.16	0.30
Age at first pregnancy (years)	16.43 ± 7.99	18 ± 2.58	17.46 ± 2.46	17.99 ± 1.85	0.001
Age at menarche (years)	11.93 ± 1.1	11.9 ± 1.01	11.95 ± 2.13	11.79 ± 0.92	0.34
Waist circumference (cm)	75.66 ± 8.79	78.46 ± 8.78	77.54 ± 8.07	77.11 ± 8.53	0.18
Fasting blood glucose (mmol/L)	5.65± 1.64	5.87 ± 2.48	6.12 ± 2.06	6.28 ± 2.62	<0.001
Triglyceride (mg/dL)	113.88 ± 81.7	115.02 ± 83.31	132.32 ± 134.54	140.93 ± 98.84	<0.001
HDL cholesterol (mg/dL)	36.99 ± 14.11	36.73 ± 14.2	38.72 ± 19.39	41.38 ± 27.72	0.003
Systolic blood pressure (mmHg)	109.5 ± 17.93	112.22 ± 17.78	116.01 ± 20.29	121.04 ± 22.46	<0.001
Diastolic blood pressure (mmHg)	72.23 ± 10.05	73.38 ± 9.32	74.85± 9.34	76.97 ± 10.14	<0.001

Abbreviation: BMI, body mass index; HDL, high-density lipoprotein

^a^Values are mean±SD, all such values.

^b^ On the basis of Mantel-Haenszel chi-square test for categorical variables and linear regression analysis for continuous variables, assigning ordinal numbers 1-4 to increasing numbers of children.

Table 2 shows odds ratio of the components of metabolic syndrome, according to increasing categories of gravity. Gravidity was positively associated with prevalence of high blood pressure both in unadjusted and multivariable adjusted model (*P* for trend = 0.01 and < 0.01, respectively). In multivariable adjusted model, compared to the lowest gravidity group (0-1), subjects with the highest gravidity (≥4) had 2.09 times higher odds of having high blood pressure. Table 3 shows odds ratio of the components of metabolic syndrome, according to increasing categories of parity. Likewise for gravidity, similar associations were found between parity and high blood pressure. Compared to lowest parity (0-1), subjects with highest parity had 1.96 times higher odds of prevalence of high blood pressure in multivariable adjusted model. Although both gravidity and parity were positively associated with high fasting blood glucose and high triglyceride in unadjusted model (*P* for trend <0.01 for both), the association disappeared after adjusting for other covariates, including demographic and lifestyle factors. Neither parity nor gravidity was associated with central obesity and low HDL cholesterol both in unadjusted and multivariable adjusted model.

**Table 2:** Odds ratio (95% confidence interval) for each component of metabolic syndrome according to gravidity.

	Number of gravidity				
	(0-1)	2	3	> = 4	Trend *P* ^b^
Central obesity (WC≥ 88 cm)					
Unadjusted	1.00	1.81 (1.04–3.17)	1.46 (0.80–2.69)	1.27 (0.69–2.35)	0.82
Multivariable adjusted^a^	1.00	1.54 (0.79–2.99)	1.10 (0.51–2.36)	1.18 (0.54–2.59)	0.89
High fasting blood Glucose (≥110 mg/dL)					
Unadjusted	1.00	0.95 (0.66–1.37)	1.42 (0.97–2.07)	1.90 (1.31–2.74)	<0.001
Multivariable adjusted^a^	1.00	0.78 (0.52–1.16)	1.05 (0.68–1.63)	1.26 (0.81–1.95)	0.15
High triglyceride (≥150 mg/dl)					
Unadjusted	1.00	1.22 (0.84–1.78)	1.34 (0.90–1.99)	1.85 (1.27–2.71)	<0.001
Multivariable adjusted^a^	1.00	1.05 (0.71–1.55)	0.98 (0.63–1.52)	1.33 (0.86–2.04)	0.23
Low HDL cholesterol (<50 mg/dl)					
Unadjusted	1.00	1.42 (0.86–2.33)	0.98 (0.59–1.62)	0.73 (0.45–1.17)	0.07
Multivariable adjusted^a^	1.00	1.48 (0.89–2.47)	1.12 (0.67–1.86)	0.88 (0.52–1.48)	0.32
High blood pressure (≥ 130|≥85 mm Hg)					
Unadjusted	1.00	1.57 (1.01–2.46)	2.72 (1.73–4.26)	4.08 (2.64–6.31)	<0.001
Multivariable adjusted^a^	1.00	1.24 (0.77–2.00)	1.66 (1.02–2.72)	2.09 (1.28–3.43)	0.01

^a^ Adjusted for age (year, continuous), BMI (kg/m^2^, continuous), marital status(currently married or others), tobacco users (ever or never), use of contraceptives (ever or never), education (illiterate, have formal education), age at first pregnancy (year, continuous).

^b^ Based on multiple logistic regression analysis, with ordinal numbers 1-4 assigned to increasing numbers of gravidity or parity.

**Table 3:** Odds ratio (95% confidence interval) for each component of metabolic syndrome according to parity.

	Number of parity				
	(0-1)	2	3	> = 4	Trend *P* ^b^
Central obesity (WC≥ 88 cm)					
Unadjusted	1.00	1.81 (1.06–3.10)	1.39 (0.77–2.51)	1.14 (0.61–2.13)	0.96
Multivariable adjusted^a^	1.00	1.48 (0.80–2.75)	1.07 (0.52–2.19)	1.01 (0.47–2.16)	0.66
High fasting blood glucose (≥110mg/dL)					
Unadjusted	1.00	0.97 (0.68–1.39)	1.68 (1.16–2.42)	1.73 (1.20–2.51)	<0.001
Multivariable adjusted^a^	1.00	0.86 (0.58–1.27)	1.27 (0.83–1.95)	1.24 (0.80–1.93)	0.12
High triglyceride (≥150 mg/dL)					
Unadjusted	1.00	1.18 (0.82–1.70)	1.33 (0.91–1.96)	1.89 (1.29–2.76)	<0.001
Multivariable adjusted^a^	1.00	1.00 (0.68–1.48)	0.96 (0.63–1.48)	1.38 (0.90–2.11)	0.16
Low HDL cholesterol (<50 mg/dL)					
Unadjusted	1.00	1.46 (0.90–2.37)	1.01 (0.62–1.65)	0.79 (0.49–1.27)	0.16
Multivariable adjusted^a^	1.00	1.51 (0.92–2.49)	1.19 (0.70–2.00)	0.94 (0.55–1.60)	0.54
High blood pressure (≥ 130|≥85 mm Hg)					
Unadjusted	1.00	1.48 (0.96–2.26)	2.87 (1.87−4.39)	3.42 (2.23–5.23)	<0.001
Multivariable adjusted^a^	1.00	1.30 (0.81–2.08)	1.82 (1.13–2.92)	1.96 (1.19–3.21)	<0.001

^a^ Adjusted for age (year, continuous), BMI (kg/m^2^, continuous), marital status(currently married or others), tobacco users (ever or never), use of contraceptives (ever or never), education (illiterate, have formal education), age at first pregnancy (year, continuous).

^b^ Based on multiple logistic regression analysis, with ordinal numbers 1-4 assigned to increasing numbers of gravidity or parity.

Table 4 shows odds ratio of prevalence of metabolic syndrome across increasing categories of gravidity and parity. Both parity and gravidity were positively associated with metabolic syndrome in unadjusted model (*P* for trend < 0.01), and additional adjustment for demographic and lifestyle factors somewhat attenuate the association. However, the association still remained significant (*P* for trend < 0.05). Subjects with the highest gravidity had 1.66 times higher odds of having metabolic syndrome than those in the lowest gravidity in multivariable adjusted model (*P* for trend = 0.02). Similarly, subjects with the highest parity had 1.65 times higher odds of having metabolic syndrome than subjects with the lowest parity (*P* for trend = 0.04).

**Table 4:** Odds ratio (95% confidence interval) of metabolic syndrome according to number of parity and gravidity.

	**Number of gravidity/parity**				
	(0-1)	2	3	> = 4	Trend *P* ^b^
**Gravidity**					
Number of cases, n (%)	39 (14.66)	72 (18.75)	68 (24.55)	94 (32.19)	
Unadjusted	1.00	1.34 (0.88–2.06)^c^	1.89 (1.22–2.93)	2.76 (1.82–4.20)	<0.01
Multivariable adjusted^a^	1.00	1.03 (0.65–1.63)	1.23 (0.76–2.01)	1.66 (1.02–2.71)	0.02
**Parity**					
Number of cases, n (%)	42 (14.74)	76 (19.54)	72 (25.62)	83 (31.44)	
Unadjusted	1.00	1.40 (0.93–2.12)	1.99 (1.31–3.04)	2.65 (1.75–4.03)	<0.01
Multivariable adjusted^a^	1.00	1.10 (0.70–1.73)	1.26 (0.78–2.05)	1.65 (1.00–2.72)	0.04

^a^ Adjusted for age (year, continuous), BMI (kg/m^2^, continuous), marital status(currently married or others), tobacco users (ever or never), use of contraceptives (ever or never), education (illiterate, have formal education), age at first pregnancy (year, continuous).

^b^Based on logistic regression analysis, with ordinal numbers 1-4 assigned to increasing numbers of gravidity and parity.

^c^ Odds ratio, 95% confidence interval in parenthesis (all such values).

Metabolic syndrome is defined as presence of at least 3 of the following criteria. Obesity component (Waist circumference ≥88 cm), high fasting blood glucose (≥110 mg/dL) or on anti-diabetic medication, high triglycerides (≥150 mg/dL), low-high-density lipoprotein (HDL) cholesterol (<50 mg/dL), high blood pressure; systolic blood pressure (SBP ≥ 130 mmHg) or diastolic blood pressure (DBP ≥85 mmHg) or on anti-hypertensive medication.

In multivariate logistic regression analyses, including the number of gravidity as a continuous variable, gravidity was significantly and positively associated with metabolic syndrome (odds ratio = 1.13, *P* = 0.04). When we considered the number of parity as a continuous variable, similar significant and positive association was found between parity and metabolic syndrome (odds ratio = 1.18, *P* = 0.03) (data not shown in Table).

Results of stratified analysis, according to menopausal status (pre-menopause or post-menopause), are presented in Table 5. The interaction between the number of parity and metabolic syndrome for menopausal women shows statistically significant results (*P* for interaction = 0.009). The number of parity was positively associated with metabolic syndrome only among pre-menopausal women (*P* for trend <0.01), but not among post-menopausal women (*P* for trend = 0.32).

**Table 5:** Odds ratio (95% confidence interval) of metabolic syndrome according to number of parity stratified by menopausal status.

	**Number of parity**				
	(0-1)	2	3	> = 4	Trend *P* ^b^
**Pre-menopausal women**					
Number of cases, n (%)	27 (11.02)	50 (14.86)	33 (17.22)	25 (25.00)	
Multivariable adjusted^a^	1.00	1.15 (0.65–2.05)^c^	1.33 (0.69–2.60)	2.62 (1.33–5.15)	<0.01
**Post-menopausal women**					
Number of cases, n (%)	15 (43.33)	26 (39.34)	39 (38.14)	58 (37.21)	
Multivariable adjusted^a^	1.00	0.83 (0.33–2.11)	0.77 (0.32–1.87)	0.68 (0.30–1.57)	0.32
***P*_interaction_^d^**		0.009			

^a^ Adjusted for age (year, continuous), BMI (kg/m^2^, continuous), marital status(currently married or others), tobacco users (ever or never), use of contraceptives (ever or never), education (illiterate, have formal education), age at first pregnancy (year, continuous).

^b^ Based on logistic regression analysis, with ordinal numbers 1-4 assigned to increasing numbers of gravidity and parity.

^c^ Odds ratio, 95% confidence interval in parenthesis (all such values)

Metabolic syndrome is defined as presence of at least 3 of the following criteria. Obesity component (Waist circumference ≥88 cm), high fasting blood glucose (≥110 mg/dL) or on anti-diabetic medication, high triglycerides (≥150 mg/dL), low-high-density lipoprotein (HDL) cholesterol (<50 mg/dL), high blood pressure; systolic blood pressure (SBP ≥ 130mm Hg) or diastolic blood pressure (DBP ≥85 mm Hg) or on anti-hypertensive medication.

^d^ Based on likelihood ratio test.

## Discussion

In the present cross-sectional study of Bangladeshi rural women, we found that the number of parity or gravidity was positively associated with metabolic syndrome after adjusting for socio-demographic, lifestyle and reproductive factors. This positive association was confined to pre-menopausal women. In addition, we also observed that of the components of metabolic syndrome, only high blood pressure shows significant positive association with the number of parity or gravidity in a multivariable adjusted model. To our knowledge, this is the first study in a South Asian population (Bangladesh), as well as from a low-income community, to address the association between parity or gravidity and metabolic syndrome.

The significant positive association between parity or gravidity and metabolic syndrome in our study is consistent with most of the previous studies [11–14]. For instance, higher parity or gravidity was positively associated with a higher prevalence of metabolic syndrome in a cross-sectional study among older Chinese women [13]. In addition, parity was positively associated with a higher prevalence of succumbing to the metabolic syndrome in an Iranian study [14]. Further, in a US study, the number of children was found to be positively associated with metabolic syndrome in women [11]. Another prospective US study also found a significantly higher prevalence of metabolic syndrome among women who had at least one non-gestational births, as compared with nulliparous women [12]. The present data confirms and extend the data of these earlier studies to a low-income South Asian country, where, paradoxically, the population has lower living conditions and body mass index but higher abdominal obesity compared to their Western counterparts and other Asians [15].

The significant positive association of parity and gravidity with metabolic syndrome in our study was confined to only pre-menopausal women. However, no significant association was found among post-menopausal women. In a previous Chinese study [13], higher parity or gravidity was associated with a consistent increase in the risk of metabolic syndrome among older women (age >50 years), where most of them may have been post-menopausal. We have no plausible reason for the significant positive association between parity or gravidity and metabolic syndrome’s existence only among pre-menopausal women in the present study, but it may be possible that other physiological changes among post-menopausal women, i.e. natural ovarian failure associated with estrogen deficiency that comes with age, may have attenuated the actual association between higher parity and metabolic syndrome among post-menopausal women.

In the present study both parity and gravidity were found to be positively associated with high blood pressure. The results of our present study are in line with those of some previous studies, where higher parity was positively associated with systolic blood pressures [20,21]. However, contrary to the present findings, most of the previous studies [11,13,14] found no clear association between parity and high blood pressure. Moreover, in a US study, gravidity was inversely associated with hypertension among both pre- and post-menopausal women [22]. In contrast, in an Italian study [23], parity was demonstrated to be independently associated with early hypertension during menopausal transition, but post-menopausal hypertension was not related with parity. In the stratified analysis of the current study, based on menopausal status, we found that parity was positively associated high blood pressure only among pre-menopausal women, with no significant association found in post-menopausal women (data not shown). The observed discrepancies between these results could be attributed, in part, to the heterogeneity in the background of the study populations. For instance, Chinese subjects were relatively older [11], whereas American subjects were much more obese [13,22], compared to our present study. It is important to note again that subjects of the present study are from rural populations and are relatively leaner, poorer, with limited or no access to resources, compared to their counterpart Asian subjects.

Like high blood pressure, parity was not significantly associated with other metabolic components (high fasting blood glucose, high triglycerides, elevated waist circumference, and low HDL cholesterol) in our present study. The relationship between births and fasting glucose levels is unclear, with some studies reporting a significant positive association [13,14], whereas others showing no clear association [11]. There have also been inconsistent reports regarding the relationship between parity and high triglycerides, low HDL cholesterol and elevated waist circumference [11,13,14]. In the face of these inconsistent data, previous studies have suggested that the observed relationships between parity and lipids are confounded or mediated by other factors, such as body weight and socio-economic status. Large prospective studies are needed to elucidate the inconsistencies in these findings.

Several biological mechanisms and lifestyle factors for the observed positive association between parity and metabolic syndrome have been suggested. First, it has been proposed that every pregnancy permanently resets ovarian function, which ultimately leads to a reduced lifetime exposure to estrogen [24] and, therefore, metabolic syndrome. Secondly, as normal pregnancy is similar to a state of insulin resistance, frequent pregnancies may result in permanent detrimental effect on lipid and glucose metabolisms [7,25,26]. Thirdly, repeated pregnancies cause excess gain in weight, weight variability or weight cycling [27,28], and will led to greater upper fat distribution and a higher prevalence of metabolic syndrome [29,30]. Finally, pregnancy and child bearing could result in subtle changes in life style and factors such as stress may not be readily measured using biological assays [31].

The major strengths of the present study include the following: a) It is a community-based survey drawn from the general population; b) the anthropometric data are generated from measurements rather than self-reporting, which is less precise; c) potential confounding variables were accounted for; d) and comparisons and associations between two birth outcomes (pregnancy or parity) and metabolic syndrome in women were examined. Despite these strengths, our study has some limitations that need to be acknowledged. First, the self-reporting of pregnancies or births may not have been reliable, likely due to recall errors. Secondly, subjects may not have been aware of or correctly recall pregnancy losses or miscarriages. However, live births are more likely to be recalled accurately than pregnancies. It is important to note that because of the similarity in results and association examined by pregnancies and births, it is likely that recall errors did not have substantially biased our results. Thirdly, although we adjusted for important confounders, the possibility of residual confounding cannot be ruled out. Finally, our study is cross-sectional and could have selection biases during case recruitment because we only examined rural women from a lower socio-economic class, and thus the results may not be generalized to the whole population of Bangladeshi women.

In conclusion, parity and gravidity were found to be positively associated with prevalence of metabolic syndrome among rural Bangladeshi women even after adjusting for potential confounding variables. The results of the present study suggest that multiparous women have increased risk of developing metabolic syndrome among women in this overpopulated country, currently facing near epidemic levels of metabolic syndrome. Further prospective studies are needed to better identify the independent lifestyle and biological factors of metabolic syndrome in relation to parity or gravidity. Such data could help in formulating effective public health policies aimed at reducing these health risks. 
